# A Qualitative Stakeholder Analysis of Avian Influenza Policy in Bangladesh

**DOI:** 10.1007/s10393-017-1285-2

**Published:** 2017-11-13

**Authors:** Kaushik Chattopadhyay, Guillaume Fournié, Md. Abul Kalam, Paritosh K. Biswas, Ahasanul Hoque, Nitish C. Debnath, Mahmudur Rahman, Dirk U. Pfeiffer, David Harper, David L. Heymann

**Affiliations:** 1 Division of Epidemiology and Public Health, The University of Nottingham, Nottingham, UK; 2 Department of Production and Population Health, Royal Veterinary College, Hatfield, UK; 3 USAID’s Preparedness and Response Project, Development Alternative Incorporated, Dhaka, Bangladesh; 4 Chittagong Veterinary and Animal Sciences University, Chittagong, Bangladesh; 5 Food and Agriculture Organisation of the United Nations, Dhaka, Bangladesh; 6 Institute of Epidemiology, Disease Control and Research, Dhaka, Bangladesh; 7 Centre on Global Health Security, Chatham House, London, UK

**Keywords:** avian influenza, poultry-related zoonoses, policy, stakeholder analysis, One Health, Bangladesh

## Abstract

Avian influenza is a major animal and public health concern in Bangladesh. A decade after development and implementation of the first national avian influenza and human pandemic influenza preparedness and response plan in Bangladesh, a two-stage qualitative stakeholder analysis was performed in relation to the policy development process and the actual policy. This study specifically aimed to identify the future policy options to prevent and control avian influenza and other poultry-related zoonotic diseases in Bangladesh. It was recommended that the policy should be based on the One Health concept, be evidence-based, sustainable, reviewed and updated as necessary. The future policy environment that is suitable for developing and implementing these policies should take into account the following points: the need to formally engage multiple sectors, the need for clear and acceptable leadership, roles and responsibilities and the need for a common pool of resources and provision for transferring resources. Most of these recommendations are directed towards the Government of Bangladesh. However, other sectors, including research and poultry production stakeholders, also have a major role to play to inform policy making and actively participate in the multi-sectoral approach.

## Introduction and Purpose

Bangladesh is a lower-middle-income country, with an agricultural-based economy (World Bank 2016a and b). Its poultry sector, both commercial and family (particularly, small extensive scavenging and extensive scavenging) poultry production, is growing rapidly (Dolberg 2008; FAO 2014). Bangladesh is one of the most densely populated countries, for both human (1072 people/km^2^) and poultry populations (1194 birds/km^2^) (World Bank 2013). Consistent with the practice in other South Asian countries, Bangladesh continues the culture of keeping animals together with people within the same house (household farms) and of live bird markets (Dolberg 2008; Gerloff et al 2016). A high proportion of all poultry products go through these live bird markets, and most of the products are sold unprocessed (Dolberg 2008). Thus, multiple factors, like rapidly growing poultry production, densely populated state, the culture of household farms and live bird markets, and selling unprocessed birds, make Bangladesh prone to zoonotic disease outbreaks, such as avian influenza. Avian influenza spreads within poultry populations, but, in some cases (such as the highly pathogenic avian influenza (HPAI) H5N1), may affect human beings and cause severe illness and death (WHO 2016a and b). Humans who have had avian influenza generally developed the infection after coming into close contact with infected birds, dead or alive. In Bangladesh, the first poultry outbreak of H5N1 was reported in 2007 (OIE 2013). Some subtypes, such as H5N1 and H9N2 which are endemic in Bangladesh, are zoonotic, and their potential for recombination and re-assortment raises concerns about their pandemic potential (Li et al 2010; Russell et al 2012; Monne et al 2013; OIE 2013). Thus, avian influenza is a major animal and public health concern in Bangladesh.

Policy development and implementation is a complex process which frequently takes place in an unstable and rapidly changing context, subject to unpredictable internal and external factors (Brugha et al 2000; Varvasovszky et al 2000). In 2006, the Government of Bangladesh adopted the first national avian influenza and human pandemic influenza preparedness and response plan to cover the period 2006–2008 (DGHS 2006). The plan provided a strategic framework for coordinating activities within and between different stakeholders (and sectors) for preparedness and response to avian and pandemic influenza in Bangladesh. The second plan was drafted in 2008, to cover the period 2009–2011, but the highest level did not approve this version (DGHS 2009). As the epidemiology of avian and pandemic influenza is evolving, it was mentioned that the plans would be periodically reviewed and revised whenever deemed necessary. However, even after a decade of development and implementation of the first plan, a stakeholder analysis was never performed, neither of the policy development process nor the actual policy. A stakeholder analysis is a systematic process of gathering and analysing information from different stakeholders in relation to the policy development process and/or the actual policy, which in turn allows the identification of opportunities for improvement/reforms (Brugha et al 2000; Schmeer 2000; Varvasovszky et al 2000). The use of qualitative stakeholder analysis as a tool has become increasingly popular in the health policy field, and this popularity reflects and recognises the central role of stakeholders in the context of policy development and implementation (Mason and Mitroff 1981; Crosby 1992; Walt 1994; Zeng et al 2017). Thus, the specific objectives of this qualitative stakeholder analysis study were to identify: (1) the reasons behind the development of the avian influenza policy in Bangladesh, (2) the sectors involved in the development of this policy, (3) the factors considered while developing this policy, (4) the future policy options to prevent and control avian influenza and other poultry-related zoonotic diseases and (5) the future policy environment that is suitable for developing and implementing such policies.

## Methods

This qualitative stakeholder analysis study consisted of two stages. At the first stage, semi-structured interviews were conducted with 23 key stakeholders from the government, international multilateral organisations, non-governmental organisations (NGOs) and trade associations in Bangladesh. Those with high-level authority and/or special knowledge were purposively selected for this purpose. Data collection and analysis proceeded simultaneously until the saturation of themes was reached (Green and Thorogood 2014). Interviews were conducted either face-to-face or through video-conferencing using a pre-developed interview guide. Questions were asked in relation to the five objectives of this study (Lindenberg and Crosby 1981; Freeman 1984). These interviews were conducted in February and March 2016, in either English or Bengali, depending on the interviewee’s preference. Each interview lasted for around 30–45 min.

At the second stage, a Chatham House roundtable was convened in Dhaka, Bangladesh, on 15 May 2016. The aim of the roundtable group discussion was to validate the findings from the first stage. The roundtable was attended by 40 key stakeholders from the government, international multilateral organisations, NGOs and trade associations in Bangladesh. Those with high-level authority and/or special knowledge were purposively selected for this purpose. The roundtable was conducted in English and under the Chatham House Rule, which states that “when a meeting, or part thereof, is held under the Chatham House Rule, participants are free to use the information received, but neither the identity nor the affiliation of the speaker(s), nor that of any other participant, may be revealed” (Chatham House 2002). This rule aims to provide anonymity to speakers in order to encourage openness and the sharing of information and is now used throughout the world as an aid to a free discussion (Chatham House 2002).

The interviews and the group discussion were noted and digitally recorded with consent. Then, they were transcribed (verbatim), translated (if necessary), anonymised and checked for accuracy. An interpretive analysis was conducted using thematic analysis (Boyatzis 1998). Transcripts were read and re-read. Initial codes were developed and applied initially to a small number of transcripts, enabling iteration of the thematic index. Coding of each transcript against the index was undertaken. Microsoft Excel 2013 was utilised in the analytic process. The study was approved by the Chittagong Veterinary and Animal Sciences University Research Ethics Committee, Bangladesh.

## Results

### Description of Participants

At the first stage, semi-structured interviews were conducted with 23 key stakeholders from the government (*n* = 8), international multilateral organisations (*n* = 6), NGOs (*n* = 5) and trade associations (*n* = 4) in Bangladesh. At the second stage, the Chatham House roundtable discussion was attended by 40 key stakeholders from the government (*n* = 14), international multilateral organisations (*n* = 13), NGOs (*n* = 9) and trade associations (*n* = 4) in Bangladesh.

### Thematic Analysis

As mentioned before, there were five study objectives (broad themes). Each of these themes is described and exemplified below.

### Reasons Behind Development of Avian Influenza Policy in Bangladesh

Stakeholders felt that the main reasons behind the development of the avian influenza policy in Bangladesh were:

- *National need* Many stakeholders reported that a decade ago, and well ahead of the first animal case of avian influenza in Bangladesh, the Government of Bangladesh prioritised the development of avian influenza policy. The main reasons given were the potential of avian influenza to negatively affect the human beings (that was, the fear of a human crisis developing) and the large and growing poultry industry in Bangladesh (that was, the threat to the national economy).
- *International pressure* Many stakeholders mentioned that a decade ago, the whole world was on high alert for an avian influenza pandemic, and the United Nations (UN) agencies (mainly, the Food and Agriculture Organisation of the United Nations (FAO) and the World Health Organisation (WHO)) encouraged every nation to develop its own avian influenza policy. The World Bank requested all the relevant ministries in each country to collaborate and prepare a single avian influenza policy for funding.

### Sectors Involved in Development of Avian Influenza Policy in Bangladesh

Stakeholders felt that a multi-sectoral approach was used to develop the avian influenza policy in Bangladesh, and they mentioned the involvement of the following sectors:

- *The Government of Bangladesh* Stakeholders mentioned that the Ministry of Health and Family Welfare, Ministry of Fisheries and Livestock, and Ministry of Environment and Forests played the main role in the development of this policy. Some of the organisations that function under these larger ministries and also contributed to the development of this policy, as reported by them, were: Department of Livestock Services (DLS), Bangladesh Livestock Research Institute (BLRI), Institute of Epidemiology, Disease Control and Research (IEDCR), National Institute of Preventive and Social Medicine (NIPSOM), Central Disease Investigation Laboratory (CDIL) and Forest Department (particularly its Wildlife and Nature Conservation Circle). At least two of these organisations, BLRI and IEDCR, are research institutions.
- *International multilateral organisations* Stakeholders stated that these organisations provided technical support and/or funds to the government during this process. The main UN agencies involved, as mentioned by them, were: FAO, WHO, World Bank and United Nations Children’s Fund (UNICEF). UNICEF, the UN’s lead communication agency in this area, led the communication efforts. In addition, the World Organisation for Animal Health (OIE) publishes relevant documents, which were used in this process. Apart from these organisations, the US Centers for Disease Control and Prevention (CDC) also provided technical support.
- *NGOs* Some of the national and international NGOs involved in the development of avian influenza policy, as reported by the stakeholders, were: Bangladesh Rural Advancement Committee (BRAC), Bangladesh Centre for Communication Programs (BCCP), International Centre for Diarrhoeal Disease Research, Bangladesh (ICDDRB), and EcoHealth Alliance. Some of these organisations also conduct research, such as BRAC and ICDDRB.
- *Trade associations* Stakeholders described these as associations which are part of the value chain, representing poultry production stakeholders such as breeders, feed millers, egg producers and poultry owners. These associations play a vital role in warning the government about current and forthcoming problems. Many of them reported that these associations were not formally involved, but some informally provided their views to the government, in particular to the Ministry of Fisheries and Livestock.

### Factors Considered while Developing Avian Influenza Policy in Bangladesh

Stakeholders felt that the main factors considered while developing the avian influenza policy in Bangladesh were:

- *International guidelines and practices* As was the situation in many other countries, many stakeholders felt that Bangladesh had limited experience in avian influenza prevention and control. They stated that international guidelines were primarily used and international practices were adapted to develop Bangladesh’s avian influenza policy.
- *Local norms, experience and evidence* Many stakeholders felt that initially, Bangladeshi social, economic and cultural conditions were not considered to any large extent during the policy development. The reason given was the country’s limited experience in avian influenza prevention and control and, thus, limited evidence on which to call. They mentioned that later in the process, local norms, experience and evidence were considered, and the policy was amended accordingly. For example, initially, the policy mentioned that commercial and family (small extensive scavenging and extensive scavenging) poultry farms within a large radius (3 km) of an affected farm would be destroyed, and this large radius was based on international guidelines. However, after the first outbreak was declared in Bangladesh, the government decided to destroy only those farms that were within a kilometre’s radius. At a later stage, the government further reduced this radius to half a kilometre in the case of family (small extensive scavenging and extensive scavenging) poultry farms and only the affected farm in the case of commercial poultry farms. Stakeholders pointed out two probable reasons behind these government decisions to reduce the radius. Firstly, the earlier decision led to a huge economic loss and caused great hardship to and fear among farmers. Secondly, many decision makers perceived the threat to human health as not very high in Bangladesh.

### Future Policy Options to Prevent and Control Avian Influenza and Other Poultry-Related Zoonotic Diseases in Bangladesh

The following policy options were recommended by the stakeholders to prevent and control avian influenza and other poultry-related zoonotic diseases in Bangladesh:

- *Base policy on the One Health concept* Many stakeholders argued that it is not considered advisable to have a single disease focused policy for each zoonotic disease, which may lose its importance within a short time period, especially in relation to other serious threats and crises.
*Stakeholders recommended developing a broad overarching policy to cover a range of zoonotic diseases, with a subsidiary plan for each zoonotic disease.*
- *Ensure policy is evidence-based* Many stakeholders felt that Bangladesh used a top-down approach when it started developing the avian influenza policy a decade ago, that was, the default use of international guidance rather than taking the risk-based approach on the basis of local (national) norms, experience and evidence. This approach was particularly used in emergency situations, which required a rapid response. Local norms (social, economic and cultural), experience and scientific evidence (especially in-country evidence) were not always considered before the policy was implemented.
*Stakeholders recommended using bottom-up approaches by the policy makers to develop policies, which should consider the local norms, experience and scientific evidence. They suggested conducting relevant and specific research to inform policy making. This includes not only quantitative and epidemiological studies, but also qualitative studies to understand the local norms and to explore the knowledge, attitude, perception, experience and behaviour of those involved in the poultry sector.*
- *Develop a policy that is sustainable* Many stakeholders felt that unsustainable policies are not beneficial. An example of an unsustainable policy given was the avian influenza compensation policy that has been used to date in Bangladesh. Initially, in order to restrict the outbreak of avian influenza, funds were available from the World Bank to cull and dispose of affected birds (of poultry farms) and to compensate farmers. This increased the reporting of any suspected cases. However, after the World Bank’s funding ceased, the government could not continue the compensation policy, possibly due to limited funds and frequent outbreaks of the disease. Many stakeholders suspected that this could have currently resulted in under-reporting of suspected cases by farmers (as there is no provision for compensation), and they might even be secretly and hastily selling infected birds in the market before news of any outbreaks spreads. Furthermore, departmental officers might also be neglecting to report any suspected cases (as the government cannot financially support the culling process).
*Stakeholders recommended that one way to create sustainable policies is through fostering a sense of ownership among those involved. In terms of economic sustainability, they suggested exploring the insurance option in Bangladesh.*
- *Review and update policy as necessary* Many stakeholders argued that in order to be useful in the longer term, it is necessary to keep policies under review and update them as necessary. They gave some examples of policies which exist to regulate the poultry trade in Bangladesh, but many have never been reviewed. An example given was the Bangladesh Animal Disease Act, 2005. They mentioned that the registration of new commercial farms is mandatory under this act. However, the rate of compliance remains questionable and, as a result, it is difficult to trace all the farms in Bangladesh.
*Stakeholders recommended reviewing and updating policies as necessary. They suggested that the review process should include stocktaking (checking which goals are achieved, or not, by the implementation of the policy and the reasons for the outcome) and consider the effectiveness, cost-effectiveness and acceptability of the policy. They suggested developing a clear set of measurable indicators that can be used to monitor the implementation success of a policy.*

### Future Policy Environment that is Suitable for Developing and Implementing Such Policies in Bangladesh

The future policy environment that is suitable for developing and implementing policies to prevent and control avian influenza and other poultry-related zoonotic diseases in Bangladesh should take into account the following points, as recommended by the stakeholders:

- *The need to formally engage multiple sectors* Many stakeholders felt that the government tried to include all the relevant sectors/organisations in the avian influenza policy making process. They argued that, however, there is no clear government policy on multi-sectoral collaboration and the current multi-sectoral collaboration in preventing and controlling poultry-related zoonotic diseases is attributable to the leadership qualities of a few senior officials.
*Stakeholders recommended establishing a physical One Health Secretariat by the Government of Bangladesh in order to sustain the collaborative work between different sectors/organisations in preventing and controlling poultry-related zoonotic diseases, to reduce the duplication of work among them, and to act quickly and efficiently. They suggested that the One Health Secretariat staff should include knowledgeable representatives from each sector—such as human health, animal health and wildlife and environment sectors of the government. They recommended that the Secretariat should establish networks with a range of stakeholders, including City Corporations that regulate local live bird markets, international multilateral organisations, NGOs, trade associations, farmers’ associations, vaccine companies and pharmaceutical companies. Stakeholders argued that this will also foster a sense of ownership and acceptability of policies across the sectors. They argued that the Secretariat can play a major role in the prevention and control of avian influenza and other poultry-related zoonotic diseases by developing, reviewing and updating these policies. This also includes identifying the evidence gap and recommending research priorities.*
- *The need for clear and acceptable leadership, roles and responsibilities* Many stakeholders stated that it was clear in the avian influenza policy examined that the Ministry of Fisheries and Livestock would lead, but that when a human infection is detected, for instance, the leadership would be shifted to the Ministry of Health and Family Welfare. They mentioned that this happened in 2008 and this dual leadership role was acceptable to both the ministries.
*Stakeholders recommended that during the development as well as the implementation of a policy, it should be clear which organisation is going to lead, and the roles and responsibilities of each organisation should be clear and acceptable. They suggested that within the One Health Secretariat, the leadership role should be transferable between its taskforces, depending on the issue (animal or human health) and needs.*
- *The need for a common pool of resources and provision for transferring resources* Stakeholders reported that although three ministries (Ministry of Health and Family Welfare, Ministry of Fisheries and Livestock and Ministry of Environment and Forests) are working together on the issue of poultry-related zoonotic diseases, there is no common pool of resources and there is no provision for inter-ministry transfer of resources in response to needs.
*Stakeholders recommended that the Government of Bangladesh should establish a common pool of resources (such as finance and manpower) for the development and implementation of these policies. They suggested that the One Health Secretariat should coordinate the transfer of resources in response to needs.*

## Discussion

A decade after development and implementation of the first national avian influenza and human pandemic influenza preparedness and response plan in Bangladesh, a qualitative stakeholder analysis was performed in relation to the policy development process and the actual policy. This study led to the recommendation of policy options to prevent and control avian influenza and other poultry-related zoonotic diseases in Bangladesh. It was recommended that the policy should be based on the One Health concept, be evidence-based, sustainable, reviewed and updated as necessary. The future policy environment that is suitable for developing and implementing these policies should take into account the following points: the need to formally engage multiple sectors, the need for clear and acceptable leadership, roles and responsibilities and the need for a common pool of resources and provision for transferring resources. Most of these recommendations are directed towards the Government of Bangladesh. However, other sectors, including research and poultry production stakeholders, also have a major role to play to inform policy making, and actively participate in the multi-sectoral approach.

Major recommendations that came up during the study were: to base the policy on the One Health concept and to establish a physical One Health Secretariat in Bangladesh. The implementation of the One Health approach to prevent and control zoonotic diseases has been advocated globally (Okello et al 2015). The term ‘One Health’ has evolved to acknowledge the close relationship between humans, animals and the natural, political and socioeconomic environments in which they coexist (One Health Initiative Task Force 2008). One Health advocates maintain that disease control as a result of inter-sectoral collaboration between the veterinary, medical and environmental sectors results in added benefits to each individual sector (Schelling et al 2005; Zinsstag et al 2005). The One Health movement has gained momentum in recent years, evolving from its roots in One Medicine to promote inter-sectoral collaboration and a ‘whole of society’ approach to global health governance (Zinsstag et al 2005 and 2007; Scoones 2010; CDC and EU 2011; Lee et al 2013). Political vigour towards zoonotic disease control under One Health appeared to escalate in 2005 after David Nabarro’s dire warning that essentially reframed the HPAI policy debate ‘from a problem of chicken farmers and hygienically inadequate markets in East and Southeast Asia to one that could affect everyone’ (BBC 2005; Scoones et al 2007). The 2008 multi-partner ‘One World, One Health’ strategic framework is a noticeable example of the ‘significant policy shift’ that took place globally at the time (FAO et al 2008; Chien 2013).

In spite of such well-intentioned initiatives, apprehension remains that the ‘big politics’ of stamping out intermittent disease outbreaks continues to dominate international health policy dialogue, neglecting different livelihoods and systems-based approaches that are arguably more relevant to developing countries or endemic situations (de Savigny et al 2004; Scoones 2010; Mwacalimba 2012). It should be noted that village poultry (small extensive scavenging and extensive scavenging family poultry, including their owners and traders) was frequently implicated in the disease transmission in the early days of the HPAI H5N1 pandemic. The pandemic had a negative impact on village poultry (small extensive scavenging and extensive scavenging family poultry) in many countries. However, with the improved understanding of the disease epidemiology, it was recognised that village poultry (small extensive scavenging and extensive scavenging family poultry) raised under extensive conditions pose relatively less of a threat than intensively raised poultry of homogenous genetic stock with poor bio-security (Alders et al 2014). Now, there is a growing awareness of the importance of facilitating socially and culturally sensitive dialogue to develop avian influenza prevention and control options. Although it is important to consider local perspectives while developing such policies, the ease of transmission of the virus should not be downplayed (Alders et al 2014). This happened in Bangladesh where the radius (of an affected farm) for the destruction of poultry farms was reduced over time making it no longer consistent with the international guidelines.

In 2012, the Ministry of Health and Family Welfare, Ministry of Fisheries and Livestock and Ministry of Environment and Forests, jointly with FAO, WHO and UNICEF, developed and endorsed a One Health strategic framework in Bangladesh (IEDCR 2012). This framework provided direction for the implementation of the One Health approach for preventing and controlling emerging, re-emerging and high-impact zoonotic diseases in Bangladesh (IEDCR 2012). However, the complete implementation of such an important framework remains questionable, even after four years of its publication. On a better note, the inter-ministerial One Health steering committee in Bangladesh, a newly established coordination body, has recently (after completion of this study) decided to establish the One Health Secretariat in Bangladesh. It has been agreed that each of the three ministries (Ministry of Health and Family Welfare, Ministry of Fisheries and Livestock and Ministry of Environment and Forests) will send one officer to the Secretariat on secondment. The terms of reference of the Secretariat have been developed through a workshop, as agreed by all the major government actors. It has been unequivocally agreed to locate the Secretariat at IEDCR, considering the fact that IEDCR has been informally playing the role of a coordinator since the inception of the One Health concept in Bangladesh. The functions of the Secretariat could be similar to that of the UK’s Human Animal Infections and Risk Surveillance (HAIRS) group, such as horizon scanning, risk assessment, risk management and risk communication (Morgan 2014), and could go beyond these tasks, such as to develop, review and update policies, as recommended by the stakeholders. The HAIRS group is a collaboration between a number of human and animal health organisations within the UK government, has been described as a major One Health initiative and has been cited as an example of how the fields of animal and human health can effectively work together on risk assessment for emerging health threats (Lightfoot et al 2013; Heymann et al 2014). Internationally, the One Health agenda tends to be dominated by veterinarians, and one of the features of the HAIRS group is the equal roles played by human and animal health practitioners. Some of the potential reasons behind the success of the HAIRS group are: a systematic approach to its work and early on agreed terms of reference, assiduously recording of discussions and decisions, effective communication and transparent risk assessment processes. Collaborative relationships are best developed ahead of a crisis and the frequent exchanges between the members of the group ensure that these relationships are maintained. It is always a balance between members being senior enough to be able to represent an organisation and having sufficient time to contribute effectively to the group. Egos, hidden agendas and professional superiority are often features of working groups, but such behaviours are rarely exhibited in the HAIRS group (Morgan 2014).

This study has a number of strengths and weaknesses. As far as we are aware, this is the first stakeholder analysis study in Bangladesh in the context of avian influenza policy. Almost all the purposively selected stakeholders participated in the study, which included a range of key stakeholders and some of them were busy, senior officials. Some of the study authors are senior officials in Bangladesh, and this helped us to gain access to these key stakeholders. In terms of generalisability, the study findings could be valid in countries having similar political, economic, social and cultural systems (such as in other South Asian countries). A single, bilingual researcher (KC) analysed the qualitative data. Thus, the data analysis could be quite subjective. However, a two-stage process was followed to validate the study findings. Single researchers can also ensure a more uniform approach in collecting qualitative data, ensuring higher reliability and more internally valid cross-comparisons of data (Brugha et al 2000; Varvasovszky et al 2000). Moreover, he was an external (non-Bangladeshi) and independent researcher, which reduced the chances of individual biases. Finally, this was a cross-sectional study, conducted over a limited period of time. The whole context of the study is subject to change over time, including stakeholders’ perception of the past, their interest or position. The political context of policy development and implementation is frequently complex and unstable, especially in many developing countries like Bangladesh, and can be subject to sudden, unexpected transformations (Brugha et al 2000; Varvasovszky et al 2000).

## Conclusion

This qualitative stakeholder analysis study specifically recommended the future policy options to prevent and control avian influenza and other poultry-related zoonotic diseases in Bangladesh, and the future policy environment that is suitable for developing and implementing such policies. Major recommendations that came up during the study were: to base the policy on the One Health concept and to establish a physical One Health Secretariat in Bangladesh. Most of these recommendations are directed towards the Government of Bangladesh. However, other sectors, including research and poultry production stakeholders, also have a major role to play to inform policy making and actively participate in the multi-sectoral approach.
